# Prevalence and predictors of medication-related emergency department visit in older adults: A multicenter study linking national claim database and hospital medical records

**DOI:** 10.3389/fphar.2022.1009485

**Published:** 2022-10-14

**Authors:** Soyoung Park, A Jeong Kim, Young-Mi Ah, Mee Yeon Lee, Yu Jeong Lee, Jungmi Chae, Ju Hyun Rho, Dong-Sook Kim, Ju-Yeun Lee

**Affiliations:** ^1^ College of Pharmacy and Research Institute of Pharmaceutical Sciences, Seoul National University, Seoul, South Korea; ^2^ Department of Pharmacy, Seoul National University Hospital, Seoul, South Korea; ^3^ College of Pharmacy, Yeungnam University, Gyeongsan, South Korea; ^4^ Department of Pharmacy, Seoul National University Boramae Medical Center, Seoul, South Korea; ^5^ Department of Pharmacy, Pusan National University Hospital, Busan, South Korea; ^6^ Department of Research, Health Insurance Review and Assessment Service, Chuncheon, South Korea; ^7^ Department of Pharmacy, Seoul National University Bundang Hospital, Seongnam, South Korea

**Keywords:** Drug-related problems, geriatrics, emergency department visit or hospitalization, inappropriate medication, adverse drug (event), underuse of medications

## Abstract

**Objectives:** Older adults are more likely to experience drug-related problems (DRP), which could lead to medication-related emergency department visits (MRED). To properly evaluate MRED, the entire history of drug use should be evaluated in a structured manner. However, limited studies have identified MRED with complete prescription records. We aimed to evaluate the prevalence and risk factors of MRED among community-dwelling older patients by linking national claims data and electronic medical records using a standardized medication related admission identification method.

**Methods:** We included older patients who visited the emergency departments of four participating hospitals in 2019. Among the 54,034 emergency department (ED) visitors, we randomly selected 6,000 patients and structurally reviewed their medical records using a standardized MRED identification method after linking national claims data and electronic medical records. We defined and categorized MRED as ED visits associated with adverse drug events and those caused by the underuse of medication, including treatment omission and noncompliance and assessed as having probable or higher causality. We assessed preventability using Schumock and Thornton criteria.

**Results:** MRED was observed in 14.3% of ED visits, of which 76% were preventable. In addition, 32.5% of MRED cases were related to underuse or noncompliance, and the rest were related to adverse drug events. Use of antipsychotics, benzodiazepines, anticoagulants, traditional nonsteroidal anti-inflammatory drugs without the use of proton pump inhibitors, P2Y12 inhibitors, insulin, diuretics, and multiple strong anticholinergic drugs were identified as predictors of MRED.

**Conclusion:** One in seven cases of ED visits by older adults were medication related and over three-quarters of them were preventable. These findings suggest that DRPs need to be systemically screened and intervened in older adults who visit ED.

## Introduction

Older adults are especially vulnerable to drug-related problems (DRPs) due to age-related changes in pharmacokinetics (Mangoni and Jackson, 2004), multimorbidity, and polypharmacy (Davies and O'Mahony, 2015). With the increase in life expectancy, the incidence of DRP in older patients has gradually increased, and hospitalization due to DRP has also increased (Veeren and Weiss, 2017). A previous study conducted in the United States in 2016 estimated that the annual cost of drug-related morbidity and mortality was equivalent to 16% of total healthcare expenditures, demonstrating the significant economic burden of DRP on the healthcare system (Watanabe et al., 2018).

To decrease the drug-related adverse health care burden, continuous identification and investigation of the contributions of adverse drug events (ADE)-related hospitalizations and associated risk factors are fundamental. A systematic review reported that the average prevalence of hospital admissions due to DRPs was 15.4%, ranging from 1.3% to 41%, and one-third of drug-related hospitalization were preventable (Ayalew et al., 2019). A retrospective study using an intervention group from a randomized controlled trial in Norway showed that approximately two out of 10 emergency department (ED) visits were drug-related, and those were mainly resulting from poor adherence and inappropriate medication use (Nymoen et al., 2022). A previous study using an administrative database from Canada showed that 0.75% of the total ED visits among older adults were associated with ADR (Wu et al., 2012).

Depending on the study population and the research methods such as outcome definition and measurement, the prevalence and the preventability of drug-related hospitalization or ED visits have been variously reported (Ayalew et al., 2019; Nymoen et al., 2022; Wallerstedt et al., 2022). Currently, most previous studies on drug-related hospitalization or ED visits have focused on ADE. However, owing to data constraints, underuse and non-adherence have not been actively studied. According to previous studies on community-dwelling adults who suffered from DRP, a total of 5%–21.6% of DRPs were due to non-adherence, and 2%–54.2% were due to underuse (Ramalho de Oliveira et al., 2010; Kovačević et al., 2017; Kari et al., 2018; Rhalimi et al., 2018).

To assess medication-related ED visits (MRED), it is essential to have a complete history of medication use. Some previous studies used claims data to investigate the prevalence of MRED (Hartholt et al., 2010; Rodenburg et al., 2011; Stausberg and Hasford, 2011; Wu et al., 2012). Due to the lack of clinical status data, such as vital signs and laboratory results, it is necessary to assume MRED only with coded diagnoses using the International Classification of Diseases (ICD) code or prescription data. On the other hand, studies conducted in single- or multi-center hospitals (Al-Arifi et al., 2014; Bénard-Laribière et al., 2015) had limitations in obtaining all of the patients’ past medical and medication history. They could estimate them by patient interviews or the records of the associated hospital visited by the patients, but those might not cover the entire prescribed medication record. Therefore, to accurately evaluate MRED, data linkages from different complementary data sources are required.

Identification of MRED highly depends on a subjective process, and it is challenging, especially in older adults, because ADEs often present as common geriatric problems or underlying diseases. To overcome this, several standardized trigger tools have been developed to evaluate MRED (Singh et al., 2009; de Boer et al., 2013; Thevelin et al., 2018). Singh et al. (2009) developed the ADR-trigger tool for the older patients in an ambulatory primary care setting, and de Boer et al. (2013) developed it for surgical patients. In addition, Thevelin et al. (2018) developed a trigger tool for the older patients that can detect medication related admissions caused by ADR, overuse, misuse, and underuse.

We aimed to evaluate the prevalence and characteristics of medication-related problems that lead to ED visits among community-dwelling older patients by linking national claim data and electronic medical records and using a standardized medication related admission identification method.

## Materials and methods

### Study settings and database

This retrospective study was conducted at four hospitals in South Korea. Hospital A (H_A_) and Hospital B (H_B_), located in the metropolitan area, are 1,779 and 1,334 bedded tertiary hospitals, respectively. Another tertiary hospital, Hospital C (H_C_), is located in the province and has 1,191 beds. The Hospital D (H_D_) is a city-run secondary hospital with 786 beds.

We used both the national claims database and the electronic medical records to overcome the limitations of each dataset. The national claims data of the Korean Health Insurance Review and Assessment Service (HIRA) include all healthcare use data of insurance beneficiaries, such as prescriptions and disease diagnoses. HIRA, an independent government-run organization, routinely collects information regarding healthcare payments for nearly 98% of South Korea’s total population. We can identify comprehensive medication use, procedures, and diagnostic codes when using the HIRA data; however, we cannot confirm the exact cause and result of a healthcare visit because there are no results such as laboratory examinations or physician assessments. We controlled for this limitation by reviewing the medical records of the study participants at ED visits.

### Study Population

The study population consisted of older patients (≥65 years old) taking medications and visiting the ED at the participating hospitals from the 01 January 2019 to the 31 December 2019. The exclusion criteria were as follows: 1) programmed rehospitalization, 2) no prescribed medication that was supposed to be taken during 1 month prior to the index date, and 3) main diagnosis of cancer, burns, open wound, or traumatic amputation. A total of 6,000 patients who visited the ED of four hospitals in 2019 were selected through simple random sampling of 54,023 patients. If two or more ED visits were identified per patient, only the first visit was included in this study, and the date of the ED visit was defined as the index date.

### Outcomes definition and measure

We defined and categorized MRED as ED visits associated with adverse drug events (MRED-ADE) and those caused by the underuse of medication, including treatment omission and noncompliance (MRED-underuse).

We developed an electronic case record form (e-CRF) with Microsoft Excel to conduct an efficient and structured record review by modifying Thevelin’s trigger tool (Thevelin et al., 2018). Before the initiation of the medical records review, patient characteristics were extracted using 1-year claims data before the index date and pre-uploaded in the e-CRF: age, sex, insurance status, medications that were supposed to be taken during 30 days prior to the visit, baseline comorbidities, and healthcare utilization. The main diagnosis and first subdiagnosis of an ED visit were also extracted based on the claims of the ED visit. Two skilled clinical pharmacists independently conducted a case review to identify MREDs. Clinical pharmacists assessed the causality, preventability, and severity of MRED-related ED visits after reviewing medical records, concurrent with pre-uploaded patient characteristics. The final results were derived through discussion of whether the evaluation results between the two evaluators were inconsistent.

The causality of DRP was evaluated using the modified version of the World Health Organization-Uppsala Monitoring center (WHO-UMC) criteria that could also assess noncompliance or treatment omission along with Klopotowska et al. (2013). We defined MRED when the causality was assessed as “Probable” or “Certain”.

Preventability was assessed using the modified Schumock and Thornton criteria (Schumock and Thornton, 1992). We modified it into 10 categories: inappropriate drug selection/therapeutic duplication, underuse, noncompliance, inappropriate dose/route/frequency, inappropriate treatment duration, lack of monitoring, allergic or non-allergic adverse drug events, toxic adverse drug events, drug-drug interactions, and drug administration errors. If more than one category is met, it is considered to be preventable. Based on the definition of preventability, all cases of MRED underuse were classified as preventable.

The severity was evaluated by the National Coordinating Council for Medication Error Reporting and Prevention (NCC MERP) (National Coordinating Council for Medication Error Reportingand Prevention, 2001). Depending on severity, it was classified into five categories: E to I. The definition of each category was as follows: E, temporary harm and required intervention; F, temporary harm and required hospitalization; G, permanent harm; H, needed intervention to maintain life; and I, caused the patient’s death.

To identify predictors associated with MRED, medication use was measured based on polypharmacy, potentially inappropriate medications (PIM) according to Beers criteria, and Screening Tool of Older Person’s Prescriptions (STOPP)/Screening Tool to Alert to Right Treatment (START) criteria (American Geriatrics Society Beers Criteria® Update Expert Panel, 2019; O'Mahony et al., 2015). Drugs used for more than 7 days a month before the index date were counted for excessive polypharmacy (10 or more drugs). We also measured the anticholinergic burden and the number of strong anticholinergic agents (3 points) according to the Korean Anticholinergic Burden Scale (K-ABS) (Jun et al., 2019).

### Analysis

Before reviewing the medical records, to accurately evaluate each patient’s records, both online and offline training sessions were conducted for pharmacists, and we evaluated the reliability between pharmacists by calculating Randolph’s values. Twenty pilot cases were divided into two groups, and 15 and 14 evaluators performed evaluations for each group. Inter-rater reliability measured using κ values for causality, preventability, and severity were adequate (0.48–0.59, 0.73–0.85, and 0.72–0.81, respectively).

Descriptive statistics were used for the prevalence and evaluation of associated factors. Multiple logistic regression analysis was performed to identify the risk factors associated with MRED: sociodemographic factors, comorbidity, healthcare utilization pattern, and medication use, including potentially inappropriate and high-risk medications. In addition, if more than 100 chief complaints or diagnoses were compiled in the MRED, a subgroup analysis was conducted. Predictors significant at an α level of 0.1 in the univariate regression analysis were entered into a multivariate regression analysis. Statistical significance was set at *p* < 0.05. All analyses were performed using SAS version 9.4 (2017 SAS Institute, Cary, North Carolina, United States).

## Results

### Characteristics of study population and prevalence of MRED

**FIGURE 1:**
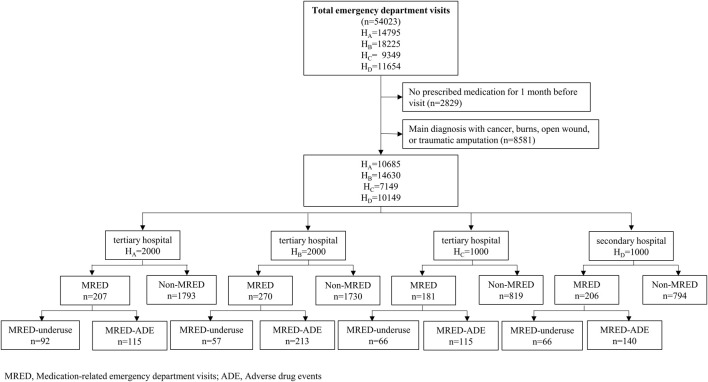
Flow diagram of identification of medication-related emergency department visits.

Overall, data from 6,000 patients were structurally reviewed (Figure 1). The characteristics of the study population are summarized in Table 1. Of the total patients, 55.1% were aged 75 years or over and 53.1% were women. Gastrointestinal disease (5,352 patients, 89.2%) was the most frequent comorbidity, followed by hypertension (4,218 patients, 70.3%) and diabetes mellitus (3,105 patients, 51.8%). Approximately one-third of the patients (2,107 patients, 35.1%) used more than 10 drugs (Table 2).

**TABLE 1:** Characteristics and medication use pattern of the study population.

Variables	Overall (*N* = 6,000)	Patients with MRED (*N* = 857)	Patients without MRED (*N* = 5,143)	*p*-Value
**Age**				
65–74	2,692 (44.9)	350 (40.8)	2,342 (45.5)	0.038
75–84	2,522 (42.0)	386 (45.0)	2,136 (41.5)	
85∼	786 (13.1)	121 (14.1)	665 (12.9)	
**Sex, Female**	3,188 (53.1)	451 (52.6)	2,737 (53.2)	0.748
**Insurance type**				
Health insurance	5,561 (92.7)	761 (88.8)	4,800 (93.3)	<0.001
Medical aid	439 (7.3)	96 (11.2)	343 (6.7)	
**Charlson comorbidity index (CCI) score**				
0	413 (6.9)	31 (3.6)	382 (7.4)	<0.001
1–2	1,714 (28.6)	208 (24.3)	1,506 (29.3)	
≥3	3,873 (64.5)	618 (72.1)	3,255 (63.3)	
**Comorbidities**				
Gastrointestinal Diseases	5,352 (89.2)	786 (91.7)	4,566 (88.8)	0.010
Hypertension	4,218 (70.3)	662 (77.2)	3,556 (69.1)	<0.001
Diabetes mellitus	3,105 (51.8)	499 (58.2)	2,606 (50.7)	<0.001
Osteoarthritis	2,686 (44.8)	407 (47.5)	2,279 (44.3)	0.083
Ischemic heart disease	2,058 (34.3)	347 (40.5)	1,711 (33.3)	<0.001
Cerebrovascular disease	1,711 (28.5)	270 (31.5)	1,441 (28.0)	0.036
Dementia	1,587 (26.5)	274 (32.0)	1,313 (25.5)	<0.001
Mood disorder	1,540 (25.7)	272 (31.7)	1,268 (24.7)	<0.001
Peripheral vascular disease	1,523 (25.4)	230 (26.8)	1,293 (25.1)	0.291
Asthma or COPD	1,478 (24.6)	225 (26.3)	1,253 (24.4)	0.234
Sleep disorder	1,102 (18.4)	185 (21.6)	917 (17.8)	0.009
Heart Failure	1,091 (18.2)	203 (23.7)	888 (17.3)	<0.001
Arrhythmia	990 (16.5)	179 (20.9)	811 (15.8)	<0.001
Neuropathy or neuralgia	902 (15.0)	154 (18.0)	748 (14.5)	0.009
Chronic kidney disease	636 (10.6)	115 (13.4)	521 (10.1)	0.004
Parkinson’s disease	436 (7.3)	65 (7.6)	371 (7.2)	0.699
Schizophrenia	174 (2.9)	43 (5.0)	131 (2.5)	<0.001
Liver failure	119 (2)	20 (2.3)	99 (1.9)	0.427
**Number of visited medical institutions (3 months)**				
0-5	5,096 (84.9)	687 (80.2)	4,409 (85.7)	<0.001
≥6	904 (15.1)	170 (19.8)	734 (14.3)	
**Number of medical institutions visits (3 months)**				
0	151 (2.5)	17 (2.0)	134 (2.6)	0.002
1–3	1,004 (16.7)	111 (13.0)	893 (17.4)	
4–6	1,229 (20.5)	164 (19.1)	1,065 (20.7)	
7–9	1,041 (17.4)	150 (17.5)	891 (17.3)	
≥10	2,575 (42.9)	415 (48.4)	2,160 (42.0)	
**History of surgery within 1 month**	412 (6.9)	49 (5.7)	363 (7.1)	0.151
**Prior ED visits within 1 month**	591 (9.9)	58 (6.8)	533 (10.4)	0.001

*Note.* COPD, chronic obstructive pulmonary disease; ED, emergency department.

**TABLE 2:** Medication use pattern of the study population.

Variables	Overall (N = 6,000)	Patients with MRED (N = 857)	Patients without MRED (N = 5,143)	*p*-Value
**Number of chronic medications**				
0–4	1,675 (27.9)	169 (10.1)	1,506 (89.9)	<0.001
5–9	2,218 (37.0)	309 (13.9)	1,909 (86.1)	
10–14	1,320 (22.0)	224 (17.0)	1,096 (83.0)	
≥15	787 (13.1)	155 (19.7)	632 (80.3)	
**Number of strong anticholinergic drugs**				
0	4,834 (80.6)	643 (75.0)	4,191 (81.5)	<0.001
1	966 (16.1)	163 (19.0)	803 (15.6)	
≥2	200 (3.3)	51 (6.0)	149 (2.9)	
**Korean anticholinergic burden scale (K-ABS)**				
0–1	3,894 (64.9)	477 (55.7)	3,417 (66.4)	<0.001
2–3	1,052 (17.5)	165 (19.3)	887 (17.2)	
4–5	616 (10.3)	118 (13.8)	498 (9.7)	
≥6	438 (7.3)	97 (11.3)	341 (6.6)	
**Number of CNS agents**				
0	3,054 (50.9)	357 (41.7)	2,697 (52.4)	<0.001
1–2	2,089 (34.8)	330 (38.5)	1,759 (34.2)	
≥3	857 (14.3)	170 (19.8)	687 (13.4)	
**Benzodiazepines with Opioids**	264 (4.4)	55 (6.4)	209 (4.1)	0.002
**Number of antihypertensive drugs**				
0–1	3,637 (60.6)	466 (54.4)	3,171 (61.7)	<0.001
2	1,452 (24.2)	230 (26.8)	1,222 (23.8)	
≥3	911 (15.2)	161 (18.8)	750 (14.6)	
**Number of oral hypoglycemic Drugs**				
0–1	4,891 (81.5)	675 (78.8)	4,216 (82.0)	0.062
2	701 (11.7)	111 (13.0)	590 (11.5)	
≥3	408 (6.8)	71 (8.3)	337 (6.6)	
**tNSAID with anticoagulants**	110 (1.8)	27 (3.2)	83 (1.6)	0.002
**tNSAID**				
Non-user	5,065 (84.4)	691 (80.6)	4,374 (85.0)	0.003
With the use of PPI	310 (5.2)	51 (6.0)	259 (5.0)	
Without the use of PPI	625 (10.4)	115 (13.4)	510 (9.9)	
**Antithrombotic therapy**				
Non-user	3,994 (66.6)	537 (62.7)	3,457 (67.2)	0.004
Acetylsalicylic acid	1,048 (17.5)	148 (17.3)	900 (17.5)	
P2Y12 Inhibitor	632 (10.5)	116 (13.5)	516 (10.0)	
DAPT	326 (5.4)	56 (6.5)	270 (5.2)	
**Diuretics**	1,328 (22.1)	248 (28.9)	1,080 (21.0)	<0.001
**Benzodiazepines**	1,006 (16.8)	198 (23.1)	808 (15.7)	<0.001
**Antidepressants**	871 (14.5)	170 (19.8)	701 (13.6)	<0.001
**Anticonvulsants**	796 (13.3)	151 (17.6)	645 (12.5)	<0.001
**Tramadol**	719 (12.0)	116 (13.5)	603 (11.7)	0.131
**Anticoagulants**	528 (8.8)	120 (14.0)	408 (7.9)	<0.001
**Opioids**	507 (8.5)	79 (9.2)	428 (8.3)	0.383
**Glucocorticoids**	430 (7.2)	71 (8.3)	359 (7.0)	0.171
**Antipsychotics**	332 (5.5)	86 (10.0)	246 (4.8)	<0.001
**Insulin**	319 (5.3)	62 (7.2)	257 (5.0)	0.007

*Note.* CNS, central nervous system; tNSAID, traditional nonsteroidal anti-inflammatory drugs; DAPT, Dual antiplatelet therapy.

A total of 1,965 ED visits contributed by medication use were identified, with certain 7.2% (141 cases), probable 36.8% (723 cases), and possible 56.0% (1,101 cases) as a result of causality assessment. The prevalence of MRED with “certain” or “probable” was 14.3% (857 patients, 864 DRP cases), of which 76% (657 cases) were found to be preventable, and those were classified into MRED-underuse (281 cases) and MRED-ADE (583 cases) (Supplementary Table S1).

### MRED-underuse

Among 281 MRED-underuse, 148 cases were due to underuse or treatment omission (52.7%) and others were noncompliance (133 cases, 47.3%). A total of 129 patients (45.9%) were hospitalized. More than half of the cases were E (141 cases, 50.2%), followed by F (115 cases, 40.9%) and G (19 cases, 6.8%), and all cases were preventable (281 cases, 100%) (Supplementary Table S2). Stroke (47 cases, 16.7%), uncontrolled hypertension (23 cases, 8.2%), hyperglycemia/ketoacidosis (22 cases, 7.8%) and cardiac arrest/ischemic disease (22 cases, 7.8%) were the main chief complaints or diagnoses of MRED-underuse.

In total, 665 medicines wer e involved in MRED-underuse. The most prevalent drug category involved in MRED-underuse was “C-Cardiovascular system” (C, 157 cases), including lipid-modifying agents (C10A, 24 cases), angiotensin II receptor blockers (C09C, 20 cases), selective calcium channel blockers (C08C, 16 cases), beta blockers (C07A, 14 cases) and high-ceiling diuretics (C03C, 13 cases). “N-Nervous system” (N, 82 cases) like anxiolytics (N05B, 13 cases) and opioids (N02A, 12 cases) were next (Table 3).

**TABLE 3:** Frequently reported drug classes related to medication-related emergency department visits.

MRED-ADE (583 cases)			MRED-underuse (281 cases)		
ATC	Description	n	ATC	Description	n
**N**	**Nervous system**	**468**	**C**	**Cardiovascular system**	**157**
N06A	Antidepressants	106	C10A	Lipid modifying agents, plain	24
N05B	Anxiolytics	88	C09C	Angiotensin II receptor blockers (ARBs), plain	20
N02A	Opioids	83	C08C	Selective calcium channel blockers with mainly vascular effects	16
N03A	Antiepileptics	60	C07A	Beta blocking agents	14
N05C	Hypnotics and sedatives	42	C03C	High-ceiling diuretics	13
N05A	Antipsychotics	34	**N**	**Nervous system**	**82**
N04B	Dopaminergic agents	25	N05B	Anxiolytics	13
N06D	Anti-dementia drugs	17	N02A	Opioids	12
**C**	**Cardiovascular system**	**268**	**A**	**Alimentary tract and metabolism**	**79**
C09C	Angiotensin II receptor blockers (ARBs), plain	69	A10B	Blood glucose lowering drugs, excl. insulins	44
C08C	Selective calcium channel blockers with mainly vascular effects	42	A10A	Insulins and analogues	12
C07A	Beta blocking agents	41	**B**	**Blood and blood forming organs**	**77**
C03A	Low-ceiling diuretics, thiazides	19	B01A	Antithrombotic agents	75
C01D	Vasodilators used in cardiac diseases	19	**M**	**Musculo-skeletal system**	**41**
C03C	High-ceiling diuretics	15	M01A	Anti-inflammatory and antirheumatic products, non-steroids	33
C08D	Selective calcium channel blockers with direct cardiac effects	13	**R**	**Respiratory system**	**35**
C01B	Antiarrhythmics, class I and III	13	R03A	Adrenergics, inhalants	11
**B**	**Blood and blood forming organs**	**166**			
B01A	Antithrombotic agents	161			
**A**	**Alimentary tract and metabolism**	**149**			
A10B	Blood glucose lowering drugs, excl. insulins	92			
A04A	Antiemetics and antinauseants	14			
A02B	Drugs for peptic ulcer and gastro-oesophageal reflux disease (GORD)	13			
A10A	Insulins and analogues	12			
**M**	**Musculo-skeletal system**	**129**			
M01A	Antiinflammatory and antirheumatic products, non-steroids	118			

*Note.* MRED, medication-related emergency department.

### MRED-ADE

A total of 583 cases (certain: 78 cases, probable: 505 cases) were counted as MRED-ADEs, of which one-third led to hospitalization (184 cases, 31.6%). Two-thirds (376 cases, 64.5%) were preventable. The detailed types of preventable MRED-ADEs were as follows: inappropriate drug selection/therapeutic duplication (260 cases, 44.6%), lack of monitoring (25 cases, 4.3%), drug-drug interaction (25 cases, 4.3%), inappropriate dose/route/frequency (23 cases, 3.9%), allergic or non-allergic ADE history (9 cases, 1.5%), inappropriate treatment duration (7 cases, 1.2%), drug administration error (7 cases, 1.2%), and toxic serum drug concentration (3 cases, 0.5%) (Supplementary Table S3). Almost two-thirds of the patients had low severity, including E (383 cases, 65.7%), F (185 cases, 31.7%), and G (7 cases, 1.2%) (Supplementary Table S2).

Overall, the ATC drug classes “N-nervous system (468 drugs),” “C-cardiovascular system (268 drugs),” and “B-blood and blood-forming organs (166 drugs)” were the most common causative drugs of MRED-ADE. The details of nervous and cardiovascular system drugs were as follows: antidepressants (N06A, 106 cases), anxiolytics (N05B, 88 cases), opioids (N02A, 83 cases), antiepileptics (N03A, 60 cases), hypnotics and sedatives (N05C, 42 cases), angiotensin Ⅱ receptor blockers (C09C, 69 cases), selective calcium channel blockers (C08C, 42 cases), and beta-blockers (C07A, 41 cases) (Table 3).

Frequent chief complaints or diagnoses for MRED-ADE were fall or fracture/hypotension/dizziness/syncope (156 cases, 26.8%), bleeding (118 cases, 20.2%), and hypoglycemia (34 cases, 5.8%). MRED-ADE-related falls or fracture/hypotension/dizziness/syncope were mostly induced by nervous system drugs (281 of 563 induced drugs), including anxiolytics (N05B, 65 drugs), hypnotics and sedatives (N05C, 32 drugs), and antipsychotics (N05A, 16 drugs). Cardiovascular system drugs (168 drugs) were also used. In addition, blood and blood-forming organs (B, 139 drugs out of 197 induced drugs) were the most common drug classes involved in MRED-ADE-related bleeding, followed by the musculoskeletal system (M, 32 drugs) and nervous system (N, 18 drugs) (Supplementary Table S4).

### Predictors of MRED

In adjusted logistic analysis, associated factors for MRED included medical aid (adjusted odds ratio (aOR) 1.42; 95% CI 1.10–1.82; compared with national health insurance), Charlson comorbidity index (CCI) score (score 1–2; aOR 1.53; 95% CI 1.03–2.28; score 3; aOR 1.74; 95% CI 1.18–2.56, compared with score 0), number of visited healthcare utilization within 3 months before the index date (≥6; aOR 1.30; 1.06–1.58; compared with <6) and prior ED visits within 1 month before the index date (aOR 0.55; 95% CI 0.41–0.74). In addition, it was associated with the participating hospitals (H_A_; aOR 0.51; 95% CI 0.41–0.64; H_B_; aOR 0.70; 95% CI 0.56–0.86; H_C_; aOR 1.1; 95% CI 0.88–1.39; compared with H_D_). Medication use and comorbidities were also related to MRED as follows: antipsychotic (aOR 1.86; 95% CI 1.41–2.46), anticoagulant (aOR 1.82; 95% CI 1.44–2.28), strong anticholinergic drugs (aOR 1.57; 95% CI 1.12–2.22), traditional nonsteroidal anti-inflammatory drugs (tNSAID) without the use of proton pump inhibitor (PPI) (aOR 1.44; 95% CI 1.15–1.81), insulin (aOR 1.39; 95% CI 1.03–1.88), diuretics (aOR 1.28; 95% CI 1.08–1.52), P2Y12 inhibitor (aOR 1.27; 95% CI 1.01–1.60) benzodiazepines (aOR 1.26; 95% CI 1.04–1.53), and ischemic heart disease (aOR 1.19; 95% CI 1.01–1.40) (Table 4). The number of central nervous system agents used (1–2; aOR 1.88; 95% CI 1.25–2.82; ≥3; aOR 2.54; 95% CI 1.51–4.29; compared with non-users) and benzodiazepines (aOR 1.84; 95% CI 1.22–2.77) were identified as medication factors related to fall or fracture/hypotension/dizziness/syncope after adjusting for other confounders. MRED-related bleeding was associated with tNSAID without the use of PPI (aOR 2.08; 95% CI 1.29–3.35), anticoagulant (aOR 7.61; 95% CI 5.19–11.16), and antiplatelet (acetylsalicylic acid; aOR 1.89; 95% CI 1.16–3.07; P2Y12 inhibitor; aOR 3.85; 95% CI 2.44–6.08; DAPT; aOR 3.49; 95% CI 1.91–6.41) (Supplementary Table S5).

**TABLE 4:** Predictors of medication-related emergency department visits.

Variables	Adjusted OR (95% CI)
**Participating hospitals**	
HA	0.51 (0.41, 0.64)
HB	0.7 (0.56, 0.86)
HC	1.11 (0.88, 1.39)
HD	reference
**Insurance**	
Health insurance	reference
Medical aid	1.42 (1.1, 1.82)
**ED visits within prior 1 month**	0.55 (0.41, 0.74)
**Number of visited medical institutions within prior 3 months**	
0-5	reference
≥6	1.3 (1.06, 1.58)
**Charlson comorbidity Index (CCI)**	
0	reference
1–2	1.53 (1.03, 2.28)
≥3	1.74 (1.18, 2.56)
**Number of strong anticholinergic drugs**	
0–1	reference
≥2	1.57 (1.12, 2.22)
**Antipsychotic**	1.86 (1.41, 2.46)
**Anticoagulants**	1.82 (1.44, 2.28)
**tNSAID without the use of PPI**	1.44 (1.15, 1.81)
**Insulin**	1.39 (1.03, 1.88)
**Diuretics**	1.28 (1.08, 1.52)
**P2Y12 Inhibitor**	1.27 (1.01, 1.6)
**Benzodiazepines**	1.26 (1.04, 1.53)
**Ischemic heart disease**	1.19 (1.01, 1.4)

*Note.* CI, confidence interval; MRED, medication-related emergency department; ED, emergency department; tNSAID, traditional nonsteroidal anti-inflammatory drugs; PPI, proton pump inhibitor.

## Discussion

This study showed that 14.3% of ED visits by older adults were medication related and over three-quarters of MREDs were preventable. These findings were in line with the results of a recent systematic review that included 16 studies investigating drug-related admissions, including ED visits (Ayalew et al., 2019; Nymoen et al., 2022). The rate of drug-related admissions was 15.4% on average, of which 44.3%–85.7% were potentially or definitely preventable. However, the current study showed a lower prevalence of MRED compared to those (42.0%) from Zerah et al. (2022)’s study that evaluated MRED visits in ambulatory older patients using Thevelin’s trigger tool. This discrepancy could be partly explained by the difference in the MRED definition. We defined MRED as when the causality assessment was certain or probable, whereas Lorene’s study defined it as possible or higher causality.

A significant proportion of MRED-ADE consequences were falls or fractures (156 cases, 26.8%), bleeding (118 cases, 20.2%), and hypoglycemia (34 cases, 5.8%), and these were mostly well-known high-alert medication-related outcomes (Sodré Alves et al., 2021; Virnes et al., 2022). Multivariate results identified high-alert medication and some potentially inappropriate medications in older adults as predictors of MRED. Antipsychotics, benzodiazepines, anticoagulants, tNSAIDs without the use of PPI, and P2Y12 inhibitors, insulin, and diuretics were associated with a higher risk of MRED. These results were similar to those of a previous study (Shehab et al., 2016). Moreover, the use of two or more strong anticholinergic agents (three points based on K-ABS) increased the likelihood of MRED by 1.57 times (95% CI 1.12–2.22) (Jun et al., 2019). Looking at the related drugs of the subgroup, which accounted for more than 100 cases in the MRED, it was found that falls and fractures, hypotension, dizziness, and syncope were related to anxiolytics, antidepressants, opioids, and antiepileptics. In addition, antithrombotic and antiplatelet drugs are the most common bleeding-related drugs, and antidiabetic drugs are associated with hypoglycemia. These results are consistent with drugs to be careful in the older patients according to the Beers criteria or STOPP/START criteria (American Geriatrics Society Beers Criteria® Update Expert Panel, 2019; O'Mahony et al., 2015).

However, the history of recent ER visits lowered the risk of MRED, which is also consistent with a previous study (Wu et al., 2012). Differences were also observed between the participating hospitals. This is because H_A_ and H_B_ are located in metropolitan areas where severely ill patients are more likely to go.

Compared to the MRED-ADE cases (34.3%), MRED-underuse cases were more classified into ‘category F’ or higher in NCC-MERP Index (49.8%). This finding, along with previous reports regarding the costs of non-adherence (Watanabe et al., 2018) suggests that more efforts should be made to address the underuse of medications and noncompliance for older patients to lower the risk of MRED underuse. Patients who had taken drugs for the cardiovascular system, nervous system, alimentary tract and metabolism, and blood and blood-forming organs were more likely to experience MRED underuse. In line with our study, Chau et al., especially investigating drugs that are related to MRED underuse, revealed that lipid-modifying agents (C10A, 2.9%) and antithrombotic agents (B01A, 2.6%) were most related (Chau et al., 2016). For some medications, such as lipid-modifying agents, it may be difficult to assume that simply not taking them for short time caused an acute disease. However, it can be assumed that significant proportion of MRED-underuse cases during 1-month prior to ED visit might have been undertreated for a long period, which might lead to acute disease.

To the best of our knowledge, this is the first study to report the prevalence and predictors of MRED in the older patients using national claims data linked to hospital medical records. One of the major strengths of the current study is that the medical records of each patient were structurally reviewed by well-trained pharmacists. In addition, using both data sets compensated for the shortcomings of each data set. The findings from this study and information regarding the contribution of DRPs to ED visits, preventability, and related factors could provide evidence for developing and implementing interventions to improve medication use, thereby preventing medication-related ED visits or hospitalization.

However, there are several limitations to be considered. First, we could not determine the patients’ use of over-the-counter drugs and drugs which were not listed in the reimbursement formulary and presumed that patients consumed only prescribed medicines if not recorded in the chart. In addition, as we assessed the only prescribed medications that were supposed to be taken during the 1-month prior to ED visit, we could not identify MRED caused by the cumulative effect of drugs used prior to the evaluation period; therefore, the MRED may have been underestimated. For this reason, results may vary depending on the lookback period for medication use. Second, due to the limitations of the retrospective study, non-adherence could only be confirmed when recorded on the chart or when patients were not prescribed the necessary drugs for a certain period. Therefore, there was the possibility of underestimation of MRED underuse. Third, some prescribed medication might not have been captured if the supplied days were not correctly recorded in the claim data as we assessed the medication use with the prescription date and their supplied days. This might be associated with the exclusion of a large number of patients (2,829 patients, 5.2%) without active prescribed medications in the 1 month prior to the ED visits. Fourth, this study included only patients who visited the ED. Thus, in future studies, it is necessary to compare them with patients who have not visited the ED to identify medication-related risk factors leading to ED visits.

In conclusion, this study showed that drug-related problems contributed to more than 14% of the total ED visits, and over three-quarters were preventable. Based on the two complementary data, the use of antipsychotics, benzodiazepines, anticoagulants, tNSAID without the use of PPI, P2Y12 inhibitors, insulin, diuretics, and two or more strong anticholinergic drugs increased the risk of MRED, and it is important to establish an appropriate prevention strategy.

## Data Availability

The raw data supporting the conclusion of this article will be made available by the authors, without undue reservation.
