# Supplementary material for: Prevalence and predictors of medication-related emergency department visit in older adults: A multicenter study linking national claim database and hospital medical records
Source: Front Pharmacol. 2022 Oct 14;13:1009485. doi: 10.3389/fphar.2022.1009485 (PMC9614024; doi:10.3389/fphar.2022.1009485)
Supplement: Supplementary file 1 [file DataSheet1.PDF]

Supplementary Table S1: Causality assessment of medication-related emergency department visits

Variables	Total	Certain	Probable	Possible
**MRED**	**1965**	**141 (7.2)**	**723 (36.8)**	**1101 (56.0)**
**MRED-underuse**	462	63 (13.6)	218 (47.2)	181 (39.2)
Stroke	87	7 (8.0)	40 (46.0)	40 (46.0)
Ischemic heart disease	48	3 (6.3)	19 (39.6)	26 (54.2)
Uncontrolled hypertension	41	5 (12.2)	18 (43.9)	18 (43.9)
Uncontrolled pain	33	4 (12.1)	17 (51.5)	12 (36.4)
COPD/Asthma	28	4 (14.3)	14 (50.0)	10 (35.7)
Hyperglycemia/Ketoacidosis	26	7 (26.9)	15 (57.7)	4 (15.4)
Heart Failure	26	6 (23.1)	13 (50.0)	7 (26.9)
Fall or Fractures/Hypotension/Dizziness/Syncope	23	3 (13.0)	8 (34.8)	12 (52.2)
Gastrointestinal diseases	23	1 (4.3)	12 (52.2)	10 (43.5)
Bleeding	23	1 (4.3)	13 (56.5)	9 (39.1)
**MRED-ADE**	1503	78 (5.2)	505 (33.6)	920 (61.2)
Fall or Fractures/Hypotension/Dizziness/Syncope	578	145 (25.1)	11 (1.9)	422 (73)
Bleeding	236	99 (41.9)	19 (8.1)	118 (50.0)
Diarrhea/Vomiting	47	14 (29.8)	6 (12.8)	27 (57.4)
Gastrointestinal diseases	45	13 (28.9)	-	32 (71.1)
COPD/Asthma	42	15 (35.7)	1 (2.4)	26 (61.9)
AV block/ Cardiac arrest	42	12 (28.6)	2 (4.8)	28 (66.7)
Constipation	40	12 (30.0)	-	28 (70.0)
Heart Failure	40	21 (52.5)	-	19 (47.5)
Hypoglycemia	40	24 (60.0)	10 (25)	6 (15)
Renal failure	35	16 (45.7)	-	19 (54.3)

MRED medication-related emergency department, COPD chronic obstructive pulmonary disease, AV block atrioventricular block.

Supplementary Table S2: Severity of medication-related emergency department visits (MRED)

Variables	Total	E	F	G	H	I
**MRED**	**864**	**524 (60.6)**	**300 (34.7)**	**26 (3)**	**2 (0.2)**	**12 (1.4)**
**MRED-underuse**	**281**	**141 (50.2)**	**115 (40.9)**	**19 (6.8)**	**2 (0.7)**	**4 (1.4)**
Stroke	47	3 (6.4)	30 (63.8)	13 (27.7)	-	1 (2.1)
Uncontrolled hypertension	23	21 (91.3)	2 (8.7)	-	-	-
Hyperglycemia/Ketoacidosis	22	8 (36.4)	12 (54.5)	1 (4.5)	-	1 (4.5)
Ischemic heart diseases	22	5 (22.7)	14 (63.6)	1 (4.5)	1 (4.5)	1 (4.5)
Uncontrolled pain	21	20 (95.2)	1 (4.8)	-	-	-
Heart Failure	19	5 (26.3)	13 (68.4)	1 (5.3)	-	-
COPD/Asthma	18	13 (72.2)	5 (27.8)	-	-	-
Bleeding	14	2 (14.3)	10 (71.4)	2 (14.3)	-	-
Gastrointestinal diseases	13	11 (84.6)	2 (15.4)	-	-	-
Fall or Fractures/Hypotension/Dizziness/Syncope	11	7 (63.6)	4 (36.4)	-	-	-
**MRED-ADE**	**583**	**383 (65.7)**	**185 (31.7)**	**7 (1.2)**	**-**	**8 (1.4)**
Fall or Fractures/Hypotension/Dizziness/Syncope	156	131 (84)	24 (15.4)	-	-	1 (0.6)
Bleeding	118	51 (43.2)	60 (50.8)	5 (4.2)	-	2 (1.7)
Hypoglycemia	34	27 (79.4)	7 (20.6)	-	-	-
Heart Failure	21	5 (23.8)	16 (76.2)	-	-	-
Diarrhea/Vomiting	20	11 (55)	9 (45)	-	-	-
COPD/Asthma	16	11 (68.8)	4 (25)	-	-	1 (6.3)
Renal failure	16	5 (31.3)	10 (62.5)	-	-	1 (6.3)
AV block/ Cardiac arrest	14	8 (57.1)	6 (42.9)	-	-	-
Urinary retention	14	11 (78.6)	3 (21.4)	-	-	-
Stroke	13	1 (7.7)	10 (76.9)	2 (15.4)	-	-

MRED medication-related emergency department, COPD chronic obstructive pulmonary disease, AV block atrioventricular block.

Supplementary Table S3: Classification of preventable medication-related emergency department visits (MRED)

Variables	Preventable
**MRED**	**657/864 (76.0)**
**MRED-underuse**	**281/281 (100)**
underuse	148 (52.7)
non-compliance	133 (47.3)
**MRED-ADE**	**376/583 (64.5)**
Inappropriate drug selection/Therapeutic duplication	260 (44.6)
Lack of monitoring	25 (4.3)
Drug-drug interaction	25 (4.3)
Inappropriate dose/route/frequency	23 (3.9)
Allergic or nonallergic adverse drug event	9 (1.5)
Inappropriate treatment duration	7 (1.2)
Drug administration error	7 (1.2)
Toxic serum drug concentration	3 (0.5)
Others	17 (2.9)

MRED medication-related emergency department.

Supplementary Table S4: Frequently reported drug classes related to medication-related emergency department visits by subgroups

ATC	Description	n	ATC	Description	n
**Fall or Fractures/Hypotension/Dizziness/Syncope**		**563**	**Bleeding**		**197**
**N**	**Nervous system**	**281**	**B**	**Blood and blood forming organs**	**139**
**N05B**	Anxiolytics	65	**B01A**	Antithrombotic agents	139
**N06A**	Antidepressants	65	**M**	**Musculo-skeletal system**	**32**
**N03A**	Antiepileptics	46	**M01A**	Antiinflammatory and antirheumatic products, non-steroids	31
**N02A**	Opioids	40	**N**	**Nervous system**	**18**
**N05C**	Hypnotics and sedatives	32	**N06A**	Antidepressants	14
**N05A**	Antipsychotics	16			
**N04B**	Dopaminergic agents	11			
**C**	**Cardiovascular system**	**168**			
**C09C**	Angiotensin II receptor blockers (ARBs), plain	48			
**C08C**	Selective calcium channel blockers with mainly vascular effects	36			
**C07A**	Beta blocking agents	27			
**C01D**	Vasodilators used in cardiac diseases	16			
**C03A**	Low-ceiling diuretics, thiazides	11			
**R**	**Respiratory system**	**44**			
**R06A**	Antihistamines for systemic use	42			
**G**	**Genito urinary system and sex hormones**	**43**			
**G04C**	Drugs used in benign prostatic hypertrophy	34			
**A**	**Alimentary tract and metabolism**	**18**			
**A04A**	Antiemetics and antinauseants	10			

Supplementary Table S5: Predictors of medication-related emergency department visits by subgroups

Variables	Adjusted OR (95% CI)	Variables	Adjusted OR (95% CI)
**Fall or Fractures/Hypotension/Dizziness/Syncope**		**Bleeding**	
**Number of CNS agents**		**tNSAID without the use of PPI**	2.08 (1.29-3.36)
0	reference	**Anticoagulant**	7.59 (5.17-11.13)
1~2	1.88 (1.25-2.82)	**Antiplatelet**	
≥3	2.54 (1.51-4.29)	Acetylsalicylic acid	1.91 (1.18-3.11)
**Benzodiazepine**	1.84 (1.22-2.77)	P2Y12 inhibitor	3.89 (2.46-6.15)
		DAPT	3.53 (1.93-6.48)

CNS central nervous system, tNSAID traditional nonsteroidal anti-inflammatory drugs, PPI proton pump inhibitor, DAPT Dual antiplatelet therapy.
